# Friction Behavior of Silver Perrhenate in Oil as Lubricating Additive for Use at Elevated Temperatures

**DOI:** 10.3390/ma12132199

**Published:** 2019-07-08

**Authors:** Junhai Wang, Ting Li, Tingting Yan, Xiaoyi Wei, Xin Qu, Shuai Yuan

**Affiliations:** School of Mechanical Engineering, Shenyang Jianzhu University, No.9 Hunnan East Road, Hunnan District, Shenyang 110168, China

**Keywords:** silver perrhenate, additive, lubrication, elevated temperatures

## Abstract

In this study, we use an aqueous solution synthesis method to prepare silver perrhenate powders and suspend them into a poly alpha olefin (PAO) base oil with polyoxyethylene octylphenyl ether. Four ball tests and ball-on-disk reciprocating mode are performed to determine how silver perrhenate performs tribologically as a lubricating additive over a wide range of temperatures. The physical and chemical properties, as well as the lubricating mechanisms of the silver perrhenate additive, are characterized via X-ray diffraction, scanning electron microscope, Fourier transformation infrared spectroscopy, Raman spectrum, and X-ray photoelectron spectroscopy. The four-ball test results demonstrate that the oil added with silver perrhenate additive is more effective than the base oil in reducing friction and improving wear resistance, and provides the best lubricating performance when at a concentration of 0.5 wt%. The reciprocating mode findings indicate that the hybrid lubricant exhibits distinctively better tribological properties than the base oil at high temperatures, and its low shear strength and chemical inertness allow for low friction at elevated temperatures. The resulting silver perrhenate layer that incorporates native superalloy oxides on the worn surface can provide lubrication by serving as a barrier that prevents direct contact between the rubbing surfaces at elevated temperatures.

## 1. Introduction

The moving parts of a machine are often subjected to extremely harsh conditions when, for example, they are used in aerospace and aviation applications [1,2,3,4,5]. The friction and wear occurring on the contacting surfaces at elevated temperatures can pose major technical challenges to the precision and service life of a machine. Oil lubricants are broadly used in modern machines due to their advantages of low friction, low wear, low noise, and sound sealability. However, their poor thermal stability makes them prone to decomposing into products that impede lubrication in high-temperature environments [6,7]. Solid lubricants are often employed at elevated temperatures to meet an array of lubrication purposes [8,9]. Nevertheless, due to their hard and brittle nature, solid lubricants exhibit a relatively high friction coefficient at low temperatures, which undermines attempts to minimize friction. New antifriction alternatives should combine multiple lubricating materials to provide efficient lubrication at high and low temperatures as well as low friction and high wear resistance at medium temperatures. The reason is that a single type of lubricant is so sensitive to temperatures that it can only reduce friction across a limited range of temperatures [10,11]. Recent years have witnessed many research efforts in the tribology community to build novel lubricating composites or modes in which two or more components are combined to enable superb tribological performance. The most promising candidate for this type of hybrid lubricant would be one that features oil and solid lubricants to achieve lubrication in a broad temperature range [12,13]. Improved hybrid lubricants are supposed to be capable of withstanding high temperatures and maintaining the smallest possible friction at low temperatures. To fabricate such a hybrid lubricant, it is critical to identify an appropriate solid lubricant that can provide effective lubrication at elevated temperatures and cause no damage to the base oil’s lubricating characteristics at low temperatures.

The lubricating action of double metal oxides under harsh working conditions has drawn considerable attention from researchers. Because they are shear susceptible and highly ductile at elevated temperatures, these double metal oxides have been widely exploited in studies focusing on solid lubricating materials [14,15,16,17]. Double metal oxides demonstrate lower Mohs hardness and greater thermal stability than individual oxides. They are therefore often used to protect contacting surfaces at elevated temperatures, either by direct preparation or tribochemical reaction over the course of friction. Zhu et al. researched how the use of barium salt affects the tribological properties of the Ni_3_Al intermetallic composite at elevated temperatures. It was found that the generation of BaCr_2_O_4_ and BaMoO_4_ plays a significant role in lubrication as the temperature increases [18]. Wang et al. investigated the tribological features of the various oxides for NiCr-WC-Al_2_O_3_ matrix composites at different temperatures. They concluded that the NiCr_2_O_4_ and NiWO_4_ formed on the composites’ worn surface at elevated temperatures lead to the reduction in both friction and wear rate [19]. Rhenium has aroused growing research attention for tribological applications due to its enhanced practicality and the deepening understanding of its properties. It has been reported that the formation of double oxides containing rhenium empowers Co-Cu-Re and Ni-Cu-Re alloys with the self-lubricating property at high temperatures, which ensures enhanced lubricating performance at elevated temperatures [20]. Owing to its softness and high thermal conductivity, silver has been recognized as a good candidate for a solid lubricant additive, and has been widely used to fabricate composites and coatings [21,22,23,24,25]. Torres et al. added Ag and MoS_2_ into the nickel-based alloy by laser cladding and discussed the role the two additives play in phase composition and microstructure [26,27]. It was revealed that these two additives keep the friction coefficient at a low level across low temperatures, and the in-situ formation of lubricant silver molybdates with a shear-susceptible microstructure helps in strengthening tribological properties at high temperatures. Yu et al. studied the tribological performance of WCN-Ag films containing different silver contents prepared by a magnetron sputtering system. The experimental results demonstrated that the presence of the Ag phase leads to a decrease in the friction coefficient of WCN-Ag films at room temperature. The Ag_2_O and AgWO_4_ oxides formed during the sliding process were found to function as lubricants at elevated temperatures [28]. According to previous studies, the silver–rhenium double oxide can be utilized as a cost-effective and efficient lubricating additive.

In this work, silver perrhenate powders are prepared using an aqueous solution synthesis method and then suspended into the poly alpha olefin (PAO) base oil via the polyoxyethylene octylphenyl ether (OP10) surfactant. The potential of silver perrhenate as a candidate oil additive for the steel/steel or ceramic/steel friction pair at different temperatures is explored by investigating its tribological performance under various test conditions. This study is expected to provide helpful insights into the lubrication mechanisms of this hybrid lubricant and may help promote its applicability as an antifriction material across a broad scope of temperatures.

## 2. Materials and Methods

### 2.1. Preparation of Materials

Pure rhenium powders (Re, ≥99.99%) were commercially supplied by Hunnan Zhonglai Tech Co., Ltd. (Zhuzhou, China). The hydrogen peroxide (H_2_O_2_, 30% concentration), silver oxide powder (Ag_2_O, ≥99.9%), and polyoxyethylene octyphenyl ether were purchased from Shanghai Macklin Biochemical Co., Ltd. (Shanghai, China). All the chemicals were utilized as received without further treatment.

The silver perrhenate powders were prepared with the following procedure. One point one two grams of rhenium powders were weighed, and then quickly added into 40 mL of hydrogen peroxide solution. The hybrid compounds were stirred vigorously for 15 min until the solution became transparent. The perrhenic acid (HReO_4_) was prepared until no bubbles could be seen. Subsequently, 0.94 g of silver oxide powders were put into freshly made HReO_4_ solution and stirred. After the reaction finished, the bottom sediments were eliminated with qualitative filter paper. Then, the resulting reactant solution was secured in a 70 °C water bath to form a thin uniform layer of material. This designed product (AgReO_4_) was transferred from the water bath to an electric oven and dried at 150 °C for 2 h. 

The commercially available PAO synthetic oil was chosen as the base fluid for its sound thermal stability, high viscosity index, high flash and ignition points, low volatility, and low pour point. The oil has several fundamental physical parameters, including a flash point of 242 °C, a pour point of −55 °C, a viscosity index of 117, and a viscosity of 30.4 mm^2^/s at 40 °C and 5.5 mm^2^/s at 100 °C. Dispersion is a key factor to consider when choosing an additive for real-world lubrication applications. Hence, an emulsifying dehydration dispersion method was utilized to obtain a lubricating mixture with sound suspension performance. For illustration purposes, 5 g of oil with 0.5 wt% AgReO_4_ additive suspended in it was prepared with the following procedure. Point zero two five grams of AgReO_4_ compounds were weighed and dissolved sufficiently in deionized water to produce a supersaturated solution. Then, 0.05 g of OP10 solution was instilled into the base oil. After a 30-min sonication process, the base oil was emulsified, producing a homogeneous mixed solution. The supersaturated aqueous solution was put into the base oil containing OP10, and then in a 130 °C oil bath for 15 min of magnetic stirring at a speed of 1000 rpm until there were no observable bubbles. At this point, the PAO base oil containing different concentrations of AgReO_4_ additive were prepared.

The phase of the designed sample was examined using an X-ray powder diffraction tester (XRD7000, Shimadzu, Kyoto, Japan), which was run at a voltage of 40 kV under Cu Kα radiation (λ = 0.154 nm) in the 10° to 90° 2θ range. Raman spectra were obtained via an XploRA PLUS confocal microscopic Raman spectroscope (HORIBA, Paris, France), with a 638 nm line solid laser acting as the excitation source to identify component and characteristic peaks. Using the FEI INSPECT-F50 scanning electron microscope (SEM, FEI, Waltham, MA, USA) that incorporates an energy dispersive spectroscopy (EDS), the sample’s morphology and composition were examined. Thermogravimetric (TG) analysis was performed using an STA 449 F5 thermal analyzer (NETZSCH, Selbu, Germany). The TG experiment was conducted in the air atmosphere at a heating rate of 10 °C/min and temperature varying from 25 °C to 1000 °C. To examine the dispersion stability of silver perrhenate in the base oil, we used a Genesys 10 ultraviolet and visible spectrophotometer (Thermo Fisher Scientific, Waltham, MA, USA) to measure the transmittances of the PAO oil containing different concentrations of silver perrhenate additive. Deionized water, with its transmittance defined as 100%, was used as the reference solution. The sample’s chemical composition was observed with a Nicolet iS5 Fourier transform infrared spectrometer (FT-IR, Thermo Scientific), and measured with four scans in the 500 cm^−1^ to 4000 cm^−1^ range at room temperature.

### 2.2. Four-Ball Tests

An MMW-1A vertical universal frictional tester (Shunmao, Jinan, China) was used to perform four-ball tests on different lubricants. The tests were designed following the GB 3142/82 standard, the Chinese equivalent to ASTM D-2783. GCr15 bearing steel balls having a diameter of 12.7 mm and a hardness varying from 59 to 61 HRC were chosen for the tests. The samples were tested at room temperature, at a rotational speed of 1450 rpm for 30 min. The impacts of the oil containing different concentrations of additive (0 wt%, 0.1 wt%, 0.3 wt, 0.5 wt%, 0.8 wt%, and 1.0 wt%) on the tribological properties were determined. Upon completion of the tests, the lower balls were cleaned with acetone for 5 min and then dried. A precision optical microscope (10 μm) was used to measure the wear scar diameter (WSD) of the lower balls. Morphologies of the wear scar were observed by using a VHE-1000 ultra depth of field microscope (Keyence, Osaka, Japan) in conjunction with the FEI INSPECT-F50 scanning electron microscope. The mean friction coefficients were calculated based on the data obtained during the tests, each repeated for three times. The increase of the oil mixture’s temperature after each test was recorded by the accompanying temperature monitoring system.

### 2.3. Evaluation of Tribological Performance across a Wide Temperature Range

**Figure 1:**
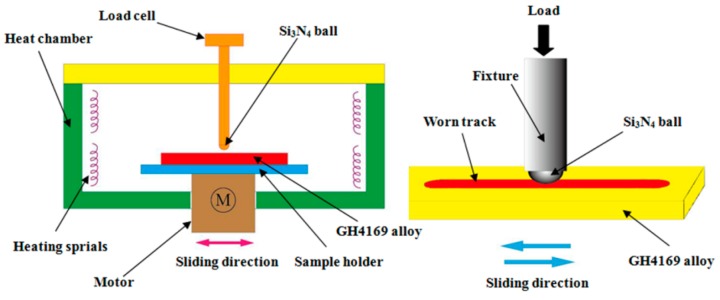
The schematic diagram of reciprocal sliding friction device.

Using the ceramic and steel materials as the friction pair, we performed the reciprocal ball-on-disk friction mode on a Rtec MFT5000 tribotester (Rtec, San Jose, CA, USA) at various temperatures. Figure 1 provides a schematic illustration of the tester integrated with a heating module. The commercially available Si_3_N_4_ ball was used as the upper sample. It came in a diameter of 6.35 mm and a surface roughness of approximately 0.02 μm. A GH4169 steel disk (Anshan Iron and Steel Group, Anshan, China) with a size of 20 mm × 20 mm × 5 mm was used as the lower sample. Its chemical composition is shown in Table 1. The steel disk was sanded with different grades of sandpapers and then polished with diamond suspension to attain a surface roughness no greater than 0.1 μm. With a sliding speed of 20 mm/s and a load of 10 N, the tests were performed at 25 °C, 100 °C, 200 °C, 300 °C, and 400 °C, each lasting for 10 min. The timely load, friction force, friction coefficient, and ambient temperature during the tests were recorded by the accompanying computer. Before testing, acetone was used to ultrasonically clean the friction pair. The purpose was to ensure the surface was free of impurities and other contaminants and has maximum stability. After that, the oil with and without the compound additive was intermittently added onto the disk surface. Analysis of the mean friction coefficients and the standard deviations was made based on the friction coefficients curves. Each test was repeated three times to ensure the attained results were repeatable and reproducible. 

Morphologies of the worn track on the disk were examined using the FEI INSPECT F50 mode scanning electron microscope. Chemical states of the worn surfaces were investigated by the combined use of an ESCALAB250 X-ray photoelectron spectroscope (XPS, Thermo Fisher Scientific, Waltham, MA, USA) and a monochromatic Al Kα X-ray source. Internal calibration of the energy scale was performed by referencing to the binding energy (284.6 eV) of the C1s peak of a carbon contaminant. The widths of the worn grooves were measured using an S3C surface profilometer (Taylor Hobson, Leicester, UK). Upon completion of the rubbing tests, Raman spectra were used to identify the composition and phase of the worn surface of the lower disk.

## 3. Results and Discussion

### 3.1. Characterization of the AgReO_4_ Additive and Suspensions

**Figure 2:**
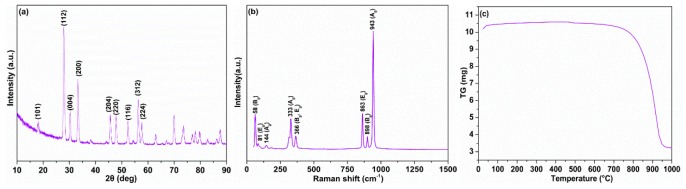
The XRD pattern, Raman spectra, and Thermogravimetric (TG) curve of as-synthesized product. (**a**) XRD pattern; (**b**) Raman spectra; (**c**) TG curve.

**Figure 3:**
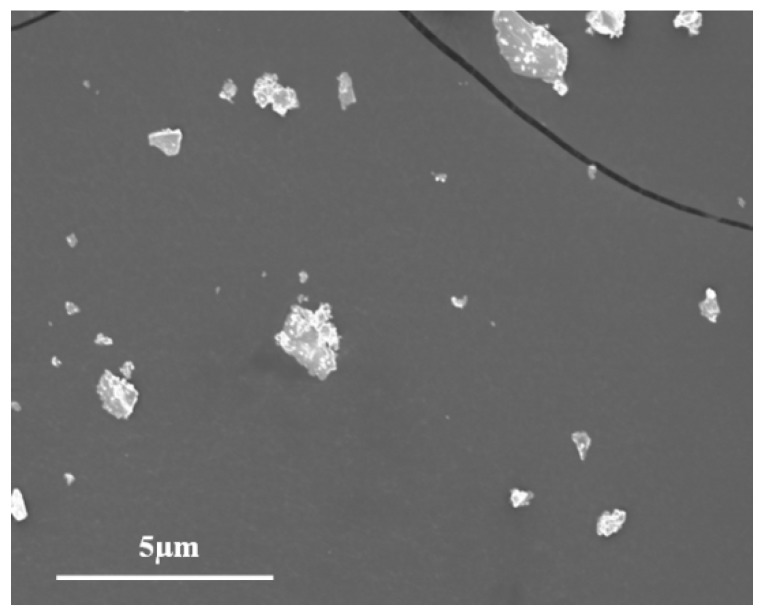
The SEM micrograph of as-synthesized product.

XRD patterns, Raman spectra, and TG curves were employed for characterizing the phase, structure, and thermal property of the synthesized product designed here. As shown in Figure 2a, all the characteristic peaks are indexed to be AgReO_4_, ideally matching with the PDF 08-0095 standard card. Intensive reflections of tetragonal AgReO_4_ are located at 27.8°, 32.3°, and 56.2°, corresponding to lattice planes of (112), (200), and (312). The XRD patterns demonstrate the ability of the aqueous solution synthesis method to prepare pure AgReO_4_ powders. Furthermore, Raman spectroscopy proves to be efficient in characterizing and determining the structure and crystallinity of double oxides. Raman spectra peaks of the designed product are located at 943, 898, 863, 366, and 333 cm^−1^. They are associated with A_g_, B_g_, E_g_, B_g_ + E_g_, and A_g_ bangs from the internal Raman mode of AgReO_4_, respectively (Figure 2b). The lattice mode at 144 cm^−1^ is assigned to the Ag rotational mode. The characteristic peak of the synthesized AgReO_4_ is located at 58 cm^−1^, resulting from the angular vibration of the B_g_ symmetric type of A_g_-A_g_ [29]. As shown in Figure 2c, the as-synthesized silver perrhenate begins to decompose at temperatures above 720 °C. This indicates that it has excellent thermostability and can be used for lubrication across a broad temperature scope. Figure 3 illustrates the morphologies of the as-product AgReO_4_ powders. We can see that the synthesized AgReO_4_ assumes a lamellar shape with a size ranging from 0.5 to 2 μm.

**Figure 4:**
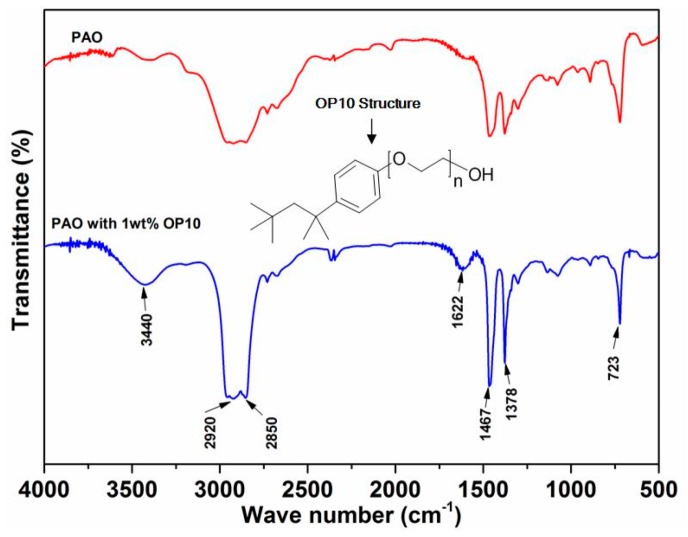
The FTIR spectra of base oil with and without added surfactant.

To understand the stability of different suspension samples, FTIR was used to identify the chemical states of the oil with and without surfactant. The FTIR spectra of pure oil and oil with 1 wt% surfactant are illustrated in Figure 4. For the oil with the surfactant, peaks are located at 2920 cm^−1^ and 2850 cm^−1^, which are attributed to the asymmetric and symmetric features of the –CH_2_– alkyl chain in the base oil. Vibration bands are observed at 1467 cm^−1^ and 1378 cm^−1^, attributable to the complexities associated with the nature of O–C=O modes [30]. Absorption peaks are observed at 1467 cm^−1^ and 1378 cm^−1^, which is a result of the symmetric and asymmetric bending vibrations of the C–H bond in the olefin oil. The bending vibration band of –CH_2_– derived from the base oil is detected at 723 cm^−1^. The abovementioned absorption peaks are also observable in the spectra of the base oil. Two distinct differences are found between the base oil and the OP10-modified one. A low, broad absorption peak is located at 3440 cm^−1^. This is attributed to the surfactant-initiated stretching vibrations of the O–H group [31]. The skeletal vibration in benzene derived from the surfactant is identified at 1622 cm^−1^ [32]. These results indicate that the OP10 surfactant provides hydroxyl functional group in the modified oil, which, in turn, creates a hydrophilic organic solvent. Moreover, the phenyl and ether groups were found to be lipophilic in OP10, which makes it easier for the OP10 oil to dissolve in the base oil.

**Figure 5:**
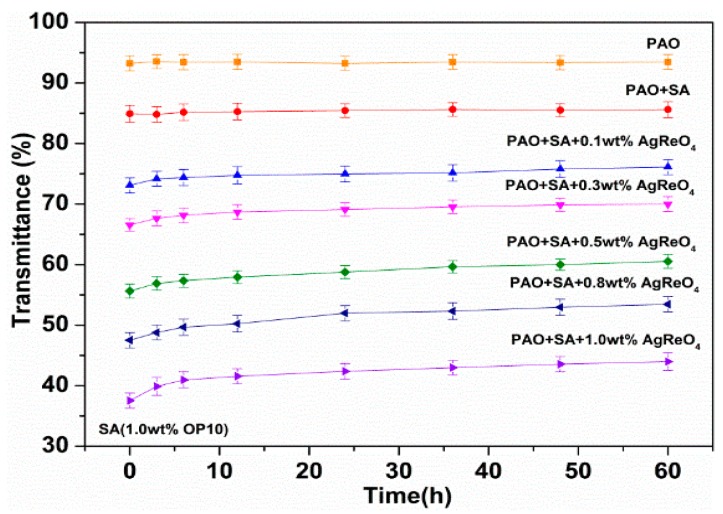
The transmittances of base oil containing various concentrations of AgReO_4_ additive with the help of SA.

Figure 5 illustrates the dispersive stability of the AgReO_4_ additive added to the modified oil. Transmittance values of the test samples decrease with the increase of AgReO_4_ concentration, demonstrating that the as-synthesized lubricating additives are suspended in the OP10-modified oil. When at a given concentration, the sample can maintain minimal transmittance increase even after dozens of standing, demonstrating its sound dispersion performance in the modified oil. This is attributable to the hydrophilically and lipophilically balanced environment in the long alkyl chains for the OP10-modified oil. The optimal lubrication performance of AgReO_4_ as a lubricating additive in the base oil is thereby guaranteed.

### 3.2. Characterization of Tribological Properties

**Figure 6:**
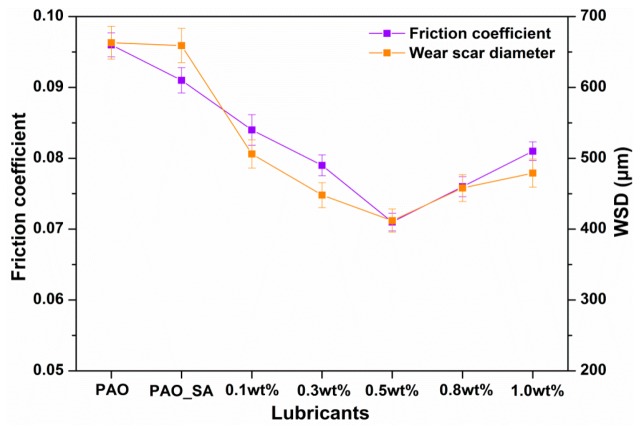
The average friction coefficients and wear scar diameter (WSD) values of varying lubricating samples.

Figure 6 shows how additive concentration affects the friction coefficient under four-ball test conditions. The oil added with AgReO_4_ additive exhibits a lower mean friction coefficient than the base oil. This indicates that the addition of the AgReO_4_ additive helps improve the lubricating performance of the base oil. Such improvement also varies with the additive concentration. The AgReO_4_ additive has excellent lubricating performance because it can easily enter into the tribological contact area under hydrodynamic action and minimizes direct contact between opposite sliding surfaces. It was also found that the mean friction coefficient decreases as the AgReO_4_ concentration increases in a range of 0.0 wt% to 0.5 wt%, and reaches its minimum, i.e., 0.071, when the concentration is 0.5 wt%. The mean friction coefficient begins to increase when the AgReO_4_ concentration exceeds the 0.5 wt% threshold. This suggests that the increase in AgReO_4_ concentration limits the additive’s capability of reducing friction and wear rate. The presence of excessive additive on the rubbing surface may hinder the supply of oil and in turn lead to increased friction force, local damage to the protective layer, and ultimately increased wear. Hence, a high concentration of AgReO_4_ additive between contacting surfaces can result in abrasion and thereby increased shear resistance and friction coefficient. Under our four-ball testing conditions, the AgReO_4_ additive is the most effective in minimizing both friction and wear when at a concentration of 0.5 wt%. Similarly, the WSD value with the addition of AgReO_4_ additive first exhibits a decrease and then an increase with the increase of additive concentration. The WSD value, when lubricated by oil with 0.5 wt% AgReO_4_ additive, is 37.9% lower than when by base oil, and also lower than when by the rest lubricating samples.

**Figure 7:**
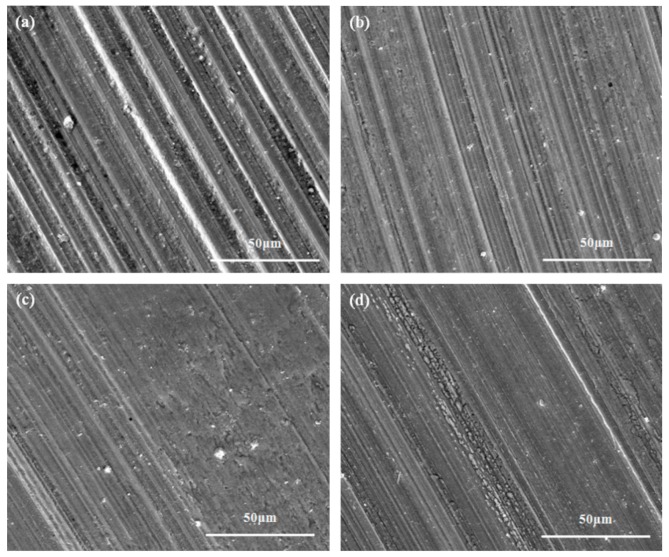
The SEM images of wear scars lubricated by different lubricating samples under four-ball test condition. (**a**) PAO; (**b**) PAO + SA + 0.3 wt% AgReO_4_; (**c**) PAO + SA + 0.5 wt% AgReO_4_; (**d**) PAO + SA + 1.0 wt% AgReO_4_.

Figure 7 shows the wear scar morphologies of the lower GGr15 steel ball lubricated by different lubricating suspensions. It can be seen that lubrication with pure PAO oil results in severe wear scars, exhibiting deep, wide grooves and plenty of wear debris. The wear mechanism involved here is the one typically experienced during abrasive wear. For lubrication with oil containing AgReO_4_ additive, however, the wear scars are much smoother, accompanied with shallow grooves and scratches. This may be because the AgReO_4_ additive is absorbed onto the rubbing surface of the steel ball, which helps in the reduction of friction. The temperature rise of the oil with different concentrations of AgReO_4_ additive is shown in Table 2. Generally, most of the dissipated energy produced by friction would be converted to heat. As such, a lower temperature rise would mean less energy loss from friction. The 0.5 wt% suspension was found to show the lowest temperature variation among all the lubricating samples. The finding here is similar to that from the friction coefficient and WSD analysis. All this further demonstrates that the AgReO_4_ compound performs well in improving the tribological properties of the base oil.

### 3.3. Tribological Characteristics over a Wide Temperature Range

**Figure 8:**
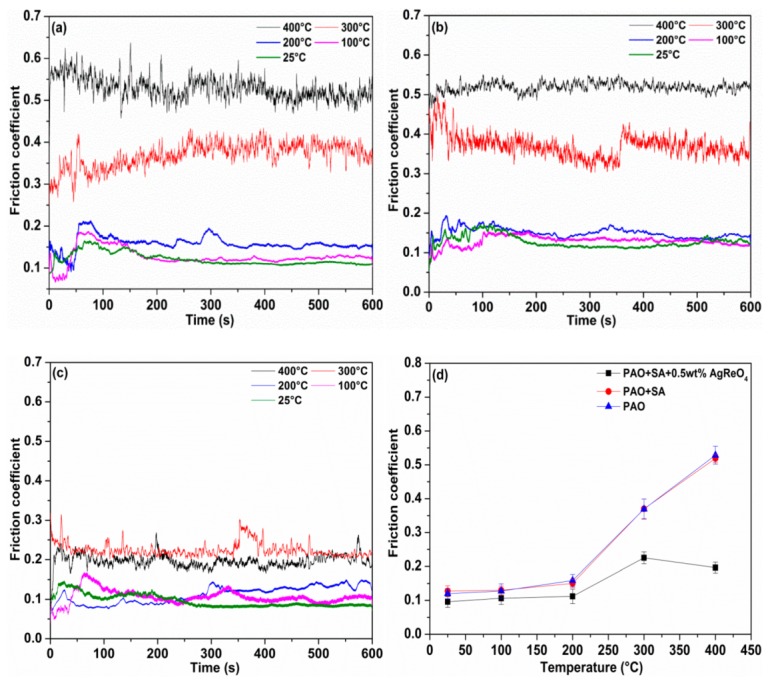
The friction coefficient curves and average friction coefficient value of worn surface lubricated by different oil mixtures at varying temperatures. (**a**) PAO; (**b**) PAO + SA; (**c**) PAO + SA + 0.5 wt% AgReO_4_; (**d**) average friction coefficient value.

The friction coefficient curves for the lubricating suspensions at different temperatures are presented in Figure 8. The friction coefficient curve for pure oil goes smoothly below 200 °C, including a mean friction coefficient of 0.121, 0.127, and 0.158 at 25 °C, 100 °C, and 200 °C, respectively. When the temperature reaches up to 300 °C, the curve exhibits a distinct fluctuation owing to oil decomposition, with a mean friction coefficient of about 0.369. This suggests high temperatures lead to decreased lubricating performance of the oil film and put the rubbing surfaces under boundary lubrication. When the temperature increases to 400 °C, the friction coefficient curve fluctuates drastically, with the mean coefficient reaching a maximum of 0.528. This indicates that the oil is completely decomposed into asphaltene, resin, and amorphous carbon [33]. The addition of surfactant has almost no effect on the lubricating performance under the experimental conditions and results in similar friction coefficient values to pure oil. This reveals that the addition of surfactant has a negligible impact on the lubricating performance of the base oil across a broad range of temperatures. From Figure 8c,d, it can be observed that the addition of AgReO_4_ additive contributes to a slight decrease in the base oil’s friction coefficient below 200 °C. At 300 °C and 400 °C, this hybrid oil exhibits a significantly lower friction coefficient than the base oil, which can be attributed to the protective layer largely generated from the AgReO_4_ additive. The friction coefficient curves imply that the addition of the AgReO_4_ additive enables lower friction at elevated temperatures, and the tribological properties of the protective layer are strongly sensitive to the testing temperature that determines the phase composition of the layer and the state of the contacting surface.

**Figure 9:**
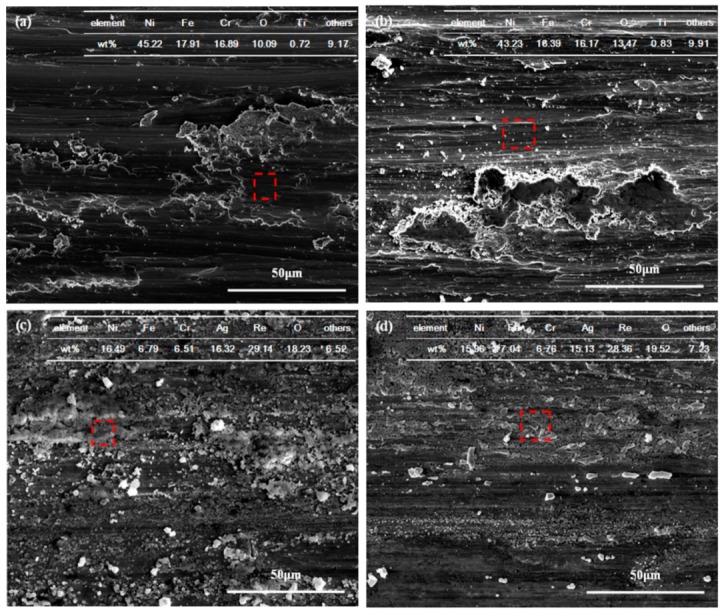
The SEM micrographs of worn surface of GH4169 alloy after 10 min sliding tests lubricated by various lubricating samples at elevated temperatures. (**a**) 300 °C, PAO; (**b**) 400 °C, PAO; (**c**) 300 °C, PAO + SA + 0.5 wt% AgReO_4_; (**d**) 400 °C, PAO + SA + 0.5 wt% AgReO_4_.

The morphologies of the worn surface after lubricated with different lubricating suspensions at various temperatures are illustrated in Figure 9. Plastic deformations, some delaminations, as well as cracks, can be observed on the worn surface (Figure 9a). Meanwhile, plenty of wear debris is detected on the worn track. The wear mechanism is characterized by micro-plowing and plastic deformation. The EDS results demonstrate the formation of O element on the worn surface. We can infer that the worn surface becomes oxidized under the joint action of ambient temperature and frictional heat. However, these oxides are brittle and easily removed in the rubbing process that follows. Hence, the native oxides formed during the rubbing test fail to function as a lubricant and make little difference in terms of friction reduction. When the temperature increases to 400 °C (Figure 9b), the base oil evaporates and is carbonized, at which point amorphous carbon is formed. Moreover, the frictional heat can lead to warmer contacting surfaces, which causes the formation of adhesive wear. Therefore, the brittle amorphous carbon derived from oil decomposition and adhesive wear is among the causes of the very high friction coefficient values. Meanwhile, the cracks caused by contact strength at the local worn area and shear failure speed up the delamination wear process. For lubrication with oil containing AgReO_4_ additive at 300 °C (Figure 9c), a thin smeared discontinuous layer is formed on the rubbing surface, accompanied by some shallow grooves and debris. Meanwhile, some slight delaminations and cracks are observed. At 400 °C (Figure 9d), the worn track is covered by a compact and relatively continuous layer, along with some native debris produced over the course of friction. According to the EDS analysis, the worn surface is mainly composed of these chemical elements: Ag, Re, O, Ni, Fe, and Cr. As a double oxide, AgReO_4_ is soft in nature and prone to being smeared on the contacting surface when applied with load. This contributes to the formation of a protective layer with some native oxide produced during the rubbing process. Moreover, this lamellar structured layer features low shear strength, which helps to reduce the friction coefficient. Therefore, the oil containing AgReO_4_ additive exhibits a dramatically lower friction coefficient than the base oil at elevated temperatures, which is attributed to the formation of the shear susceptible layer. 

**Figure 10:**
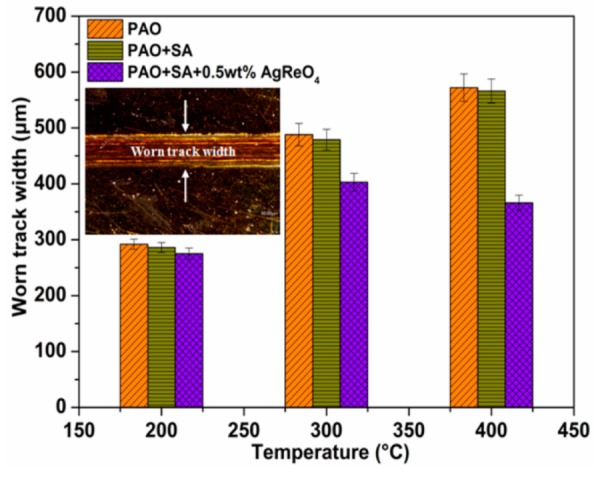
The mean width values of worn tracks lubricated by different lubricants at various temperatures.

Variations in the mean worn track width after lubrication by the oil containing silver perrhenate additive are presented in Figure 10. We see that the width of the worn track after lubrication by the base oil and the oil with SA alone increases as the temperature rises. This can be explained by the fact that the increased temperature results in oil lubrication failure and low local hardness of the superalloy and thus, the formation of noticeable materials removal and plastic deformations. At 200 °C, the worn surface has a slightly lower width when lubricated by oil with AgReO_4_ additive than when by the other two samples. However, its wear deformation is exacerbated by the temperature rise when lubricated by base oil, indicating that the oil is decomposed and cannot provide effective and consistent lubrication. The worn track reaches its maximum width at 400 °C, basically the same as the friction coefficient. Compared to lubrication with the base oil, the worn track when lubricated by oil with silver perrhenate shows a 17.4% and 36.1% lower width at 300 °C and 400 °C, respectively. This comparison indicates that the sliver perrhenate addictive can offer lubrication with low shear when pure oil lubrication fails. Furthermore, sliver perrhenate is prone to be deposited on the tribo-surface, which prevents direct contact between the ceramic/steel pairs at elevated temperatures.

**Figure 11:**
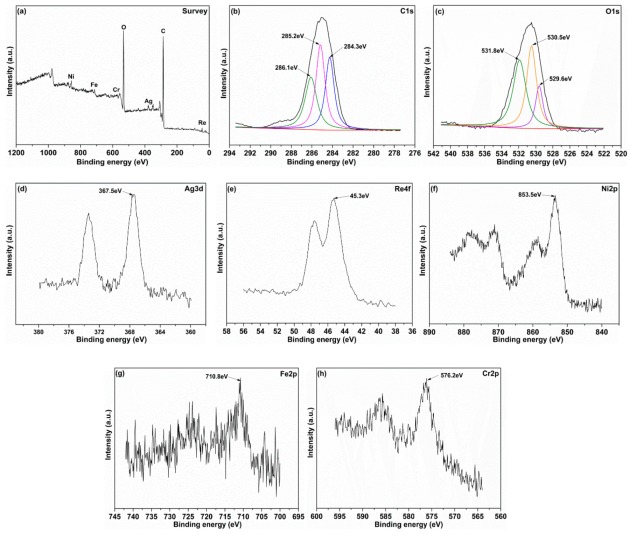
The XPS spectra of typical elements on the worn track after friction test lubricated by oil with 0.5 wt% AgReO_4_ additive at 400 °C. (**a**) Survey; (**b**) C1s; (**c**) O1s; (**d**) Ag3d; (**e**) Re4f; (**f**) Ni2p; (**g**) Fe2p; (**h**) Cr2p.

**Figure 12:**
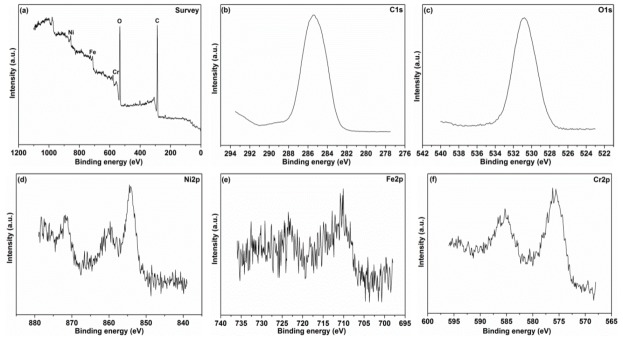
The XPS analysis of some elements on the worn track after friction test lubricated by pure PAO oil at 400 °C. (**a**) Survey; (**b**) C1s; (**c**) O1s; (**d**) Ni2p; (**e**) Fe2p; (**f**) Cr2p.

To better understand the friction reduction and lubricating mechanism of the AgReO_4_ additive in PAO, XPS was used to investigate the chemical compositions on the worn surface lubricated with the hybrid lubricating suspension at 400 °C under a load of 10 N. The survey spectra assigned to peak identification and the specific spectra of several typical elements are shown in Figure 11. Some typical bonding energies can be detected from the survey spectra, including C1s, O1s, Ag3d, Re4f, Ni2p, Fe2p, and Cr2p (Figure 11a). As shown in Figure 11b, the C1s signal consists of three main peaks. The peak located at 284.3 eV is attributed to the C–H bond in the base oil, the one at 285.2 eV to the C–O bond in the oil added with surfactant, and the one at 286.1 eV to the C–C bond in the organic substance and some contaminants introduced by the rubbing test [34]. The O1s spectrum consists of three peaks at 529.6 eV, 530.5 eV, and 531.8 eV, corresponding to ReO_4_^−^, oxygen in native superalloy oxides and oxygen in the organic substance [35]. A strong Ag3d characteristic peak is located at 367.5 eV, which is associated with the chemical state of Ag^+^. A Re4f peak is located at 45.3 eV, corresponding to the characteristic peak of Re^7+^. An intensive binding energy peak of Ni2p assigned to the nickel oxide is detected at 853.5 eV. The Fe2p peak at 710.8 eV corresponds to the chemical state of the iron in Fe_2_O_3_ phase [36]. The Cr2p peak is detected at 576.2eV, which is assigned to the production of chromium oxides during the rubbing test. The existence of native oxides on the worn surface is attributed to the oxidation during the rubbing test. The above XPS results suggest that a protective layer is deposited on the contacting surface lubricated with AgReO_4_ additive. This layer consists of AgReO_4_ and some native superalloy oxides produced during the friction process, and the AgReO_4_ additive is responsible for the reduction of friction at high temperatures. Additionally, the XPS spectra of the superalloy disk lubricated by pure PAO at 400 °C is also presented in Figure 12. The peaks of Ni, Fe, and Cr elements associated with oxidation state were detected, which suggested that the native oxides formed from the superalloy during the high temperature test made little contribution to the minimizing friction.

**Figure 13:**
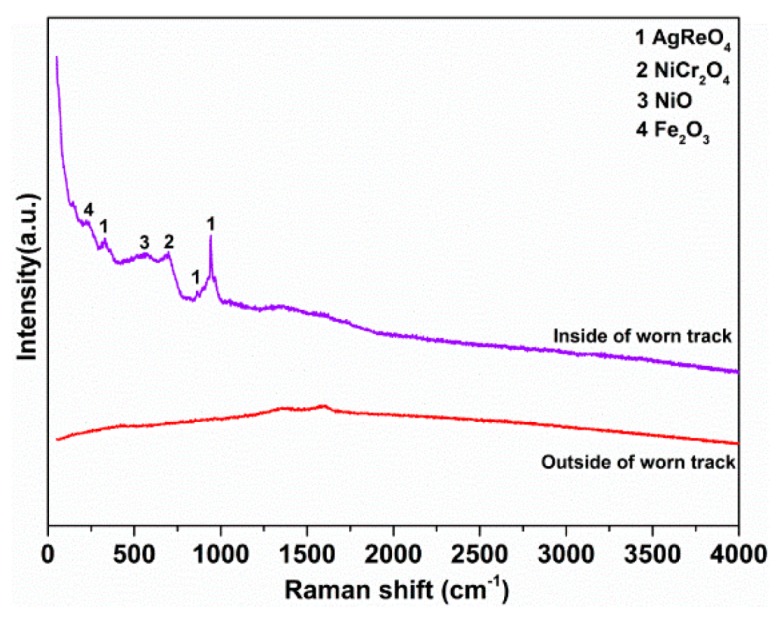
The Raman spectra of the worn surface after sliding friction test lubricated with oil containing 0.5 wt% AgReO_4_ additive at 400 °C.

The Raman spectra offer us better insights into the local phase composition inside and outside the worn track. As shown in Figure 13, a narrow peak is located at 933.2 cm^−1^, which is associated with the sliver perrhenate phase. Meanwhile, NiO, Fe_2_O_3_, and NiCr_2_O_4_ are detected on the worn track surface. This indicates that the protective layer is composed of oxides that underwent the tribochemical reaction [37,38]. Among these oxides, NiCr_2_O_4_ should be derived from the oxidation of nickel in the superalloy substrate and the subsequent reaction with chromium oxides at elevated temperatures during friction. The oxides NiO, Fe_2_O_3_, and NiCr_2_O_4_ can enhance the strength and hardness of the layer, which helps prevent furrowing wear caused by asperities on the contacting surfaces. Additionally, the soft AgReO_4_ compound can provide low shear strength and enhanced plasticity for the layer. Thus, the combination of the AgReO_4_ compound with native superalloy oxides results in improved friction-reduction performance and wear resistance.

**Figure 14:**
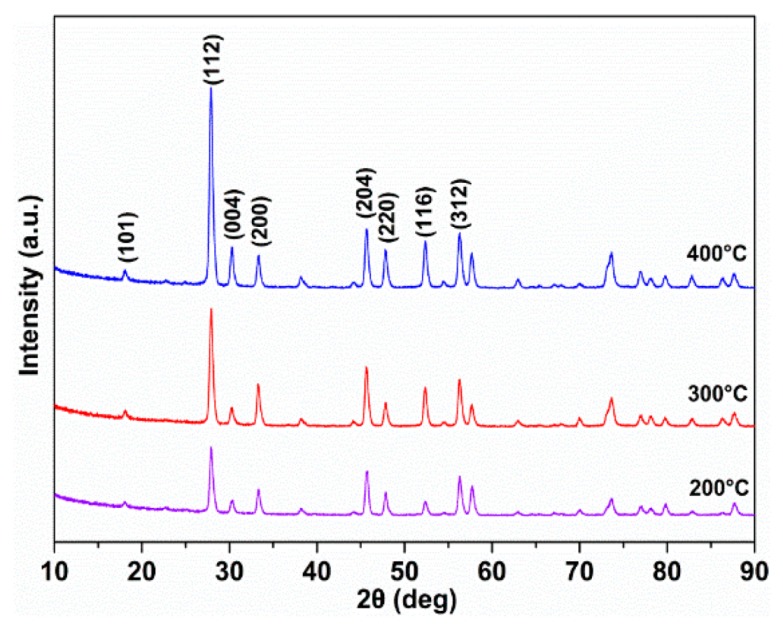
The XRD patterns of silver perrhenate powder after heat treatment for 15 min at different temperatures.

Based on the crystal chemistry theory, the tribological performances of double oxides at elevated temperatures are largely affected by the ionic potential difference [39]. The AgReO4 compound proposed here can be regarded as a double oxide consisting of two individual oxides: Ag_2_O and Re_2_O_7_, with an ionic potential of 0.8 and 12.5, respectively. Generally, the larger the ionic potential difference, the lower the melting point and the softer the double oxide will be. This explains why the AgReO_4_ compound is effective in improving the lubricating performance of base oil at a low temperature range [40]. As the ambient temperature increases, the AgReO_4_ compound becomes even softer and prone to being smeared on the worn surface, which enables low friction at high temperatures. In addition, significant ionic potential difference allows a double oxide to have a highly stable compound structure, which helps lower the attraction across the sliding contact interface. This means a lower adhesive force between the rubbing surfaces and thus minimized friction. Figure 14 shows the XRD patterns of the sliver perrhenate addictive after being heat-treated for 15 min at elevated temperatures. We see that the sliver perrhenate additive managed to keep its original phase and crystal structure at room temperature despite the increased temperatures. This suggests that it has good thermal stability and chemical durability before melting, which is a promise of its practical applicability as a high-performance lubricating additive. 

## 4. Conclusions

In this study, the influence of silver perrhenate as a lubricating additive on the tribological performances of PAO base oil is experimentally investigated under different test conditions. Here is a summary of the major new findings that we have:

(1) Compared to pure oil, the PAO base oil mixed with various concentrations of AgReO_4_ lubricating additive performs better in friction reduction and wear resistance. The four-ball test results show that the best lubricating performance is achieved when the AgReO_4_ additive is at a concentration of 0.5 wt%.
(2) The reciprocal tribological tests demonstrate that the oil added with the AgReO_4_ additive enables significantly lower friction coefficient and much higher wear resistance at high temperatures than base oil alone. Additionally, the former offers similar levels of friction reduction to the latter at low temperatures.
(3) The combination of the AgReO_4_ additive and the native oxides produced during friction results in a protective layer, which helps improve tribological performances by preventing direct contact between the friction pair at elevated temperatures. The AgReO_4_ compound’s outstanding lubrication performance is also attributed to its low shear strength and good thermal stability resulting from its significant ionic potential difference. All these results demonstrate that AgReO_4_ can be a highly promising candidate lubricating additive for addressing lubricating needs across a broad range of temperatures.

## Figures and Tables

**Table 1:** Chemical composition of GH4169 superalloy (wt%).

Specimen	C	Co	Mo	Al	Ti	Cr	Ni	Fe
GH4169	≤0.08	≤1.0	2.8–3.3	0.3–0.7	0.75–1.15	17–21	50–55	Bal.

**Table 2:** The temperature rising of different lubricating samples after the four-ball test.

Samples	PAO	PAO + SA	0.1 wt%	0.3 wt%	0.5 wt%	0.8 wt%	1.0 wt%
Starting temperature (°C)	23.6	24.5	24.1	25.2	24.8	25.4	24.3
Final temperature (°C)	59.8	59.1	57.3	54.8	52.2	55.8	57.1
Temperature variation (°C)	36.2	34.6	33.2	29.6	27.4	30.4	32.8
